# Web-Based Perspectives of Deemed Consent Organ Donation Legislation in Nova Scotia: Thematic Analysis of Commentary in Facebook Groups

**DOI:** 10.2196/38242

**Published:** 2022-09-14

**Authors:** Alessandro R Marcon, Darren N Wagner, Carly Giles, Cynthia Isenor

**Affiliations:** 1 Health Law Institute Faculty of Law University of Alberta Edmonton, AB Canada; 2 Nova Scotia Health Halifax, NS Canada

**Keywords:** organ donation, organ transplantation, deemed consent, presumed consent, social media, Facebook, public perceptions, public policy, thematic analysis

## Abstract

**Background:**

The Canadian province of Nova Scotia recently became the first jurisdiction in North America to implement deemed consent organ donation legislation. Changing the consent models constituted one aspect of a larger provincial program to increase organ and tissue donation and transplantation rates. Deemed consent legislation can be controversial among the public, and public participation is integral to the successful implementation of the program.

**Objective:**

Social media constitutes key spaces where people express opinions and discuss topics, and social media discourse can influence public perceptions. This project aimed to examine how the public in Nova Scotia responded to legislative changes in Facebook groups.

**Methods:**

Using Facebook’s search engine, we searched for posts in public Facebook groups using the terms “deemed consent,” “presumed consent,” “opt out,” or “organ donation” and “Nova Scotia,” appearing from January 1, 2020, to May 1, 2021. The finalized data set included 2337 comments on 26 relevant posts in 12 different public Nova Scotia–based Facebook groups. We conducted thematic and content analyses of the comments to determine how the public responded to the legislative changes and how the participants interacted with one another in the discussions.

**Results:**

Our thematic analysis revealed principal themes that supported and critiqued the legislation, raised specific issues, and reflected on the topic from a neutral perspective. Subthemes showed individuals presenting perspectives through a variety of themes, including compassion, anger, frustration, mistrust, and a range of argumentative tactics. The comments included personal narratives, beliefs about the government, altruism, autonomy, misinformation, and reflections on religion and death. Content analysis revealed that Facebook users reacted to popular comments with “likes” more than other reactions. Comments with the most reactions included both negative and positive perspectives about the legislation. Personal donation and transplantation success stories, as well as attempts to correct misinformation, were some of the most “liked” positive comments.

**Conclusions:**

The findings provide key insights into perspectives of individuals from Nova Scotia on deemed consent legislation, as well as organ donation and transplantation broadly. The insights derived from this analysis can contribute to public understanding, policy creation, and public outreach efforts that might occur in other jurisdictions considering the enactment of similar legislation.

## Introduction

### Background

In 2019, the Canadian province of Nova Scotia became the first jurisdiction in North America to pass legislation for organ donation instituting deemed consent, otherwise commonly known as presumed consent or opt out [1]. Within Canada, Nova Scotia has relatively high rates of organ donation [2]; however, both the province and Canada as a whole have rates lower than many other comparable regions and nations [3]. Several jurisdictions within Canada and abroad have sought to establish deemed consent donation laws to remedy organ and tissue donation shortfalls but have faced considerable criticism about the effectiveness and public reception of such proposed laws [4,5].

Studies have shown that deemed consent legislation alone does not necessarily rectify organ donation shortages [2,6,7]. Canadian Blood Services have clearly articulated the key elements for successful deceased organ donation systems within 6 significant foundational concepts, and legislation is only 1 of these 6 concepts [8]. Crucial factors for improving donation rates include properly functioning donation registries, ethical organ allocation systems, and context-sensitive donation laws [4,7,9]. In the past, some nations that instituted deemed consent laws, including Singapore, Brazil, and Chile, did not successfully increase donation and transplantation rates following legislation changes [10]. Others, such as Wales and the Netherlands, are observing an increase in donation and transplantation rates [11]. Importantly, in the context of Wales’s success, the United Kingdom has a well-established donation system infrastructure to support legislative changes [11]. The efficiency of any donation consent model depends on ancillary factors such as instilling trust in health care systems, accommodating next of kin, and creating effective public outreach [10].

### Objectives

In response to the new legislation in Nova Scotia, Health Canada has funded a program of research, Legislative Evaluation: Assessment of Deceased Donation Reform, to evaluate “the implementation process and full impact of the deceased organ donation legislation and the health system transformation” and to “inform future legislative or administrative changes to donation and transplantation in other jurisdictions” [12]. Our research contributes to the Legislative Evaluation: Assessment of Deceased Donation Reform program by examining web-based public discussions on the legislative changes in Nova Scotia. Understanding web-based public perspectives is valuable as social media can influence how the public learns about, thinks about, and acts on health topics [13-15].

We observed a substantial number of discussions on Nova Scotia’s deemed consent organ policy on Facebook. Facebook is a key platform for sharing views, exchanging information, and seeking advice about personal health actions and decisions, including at the intersection of political decisions with health ramifications [16-18]. Facebook, similar to many other social media platforms, involves community formation and group connections [19]. Numerous studies have shown how belonging to health-related Facebook groups can provide emotional support and increase social connectivity for participants [14,20]. Unlike more anonymous platforms, such as Reddit or newspaper comments sections, Facebook users commonly operate through personal profiles, which means that their activities are often seen by family and friends [21]. Research shows how Facebook users typically only follow, and participate in, a few pages in their Facebook activities [22], which demonstrates the sociological understanding of “homophily”—where people interact more with others similar to themselves [23]. Recent research has used “homophily” ideas to interpret social media interactions, showing how similar web-based interactions can strengthen ties between individuals [24]. Further research in health contexts, particularly during the COVID-19 pandemic, has shown how group formation around political or ideological lines can play an influential role in shaping perspectives and informing decisions [25], whereas other projects have demonstrated that scientific literacy and cognitive sophistication are also key drivers [26].

Facebook use is high among Canadians [27], and research shows that many Canadians use Facebook to access news stories [28]. Although research on the Canadian public demonstrates that Canadians do not commonly trust the information they come across on social media [29], it also shows that Canadians have high levels of trust in friends, family [30], and those in their local communities [31] and are willing to be persuaded by convincing arguments from individuals they trust [28]. Although organ donation is a relatively niche topic, certain Nova Scotian Facebook groups had lively discussions offering many public perspectives about the legislated changes to organ donation. However, Facebook can be a breeding ground for misinformation [32], which has raised concerns about the kinds of information with which users engage [18,33].

Observing and analyzing the deemed consent discourse in Nova Scotian Facebook groups allowed us to observe public perspectives, including how others responded to sentiments and opinions, including misinformation. Our research incorporated the user responses to Facebook posts, namely comments, replies, shares, and emoji reactions (eg, “Likes”) [34]. Research shows how emoji reactions play an important communicative role on Facebook, helping forge connectedness and social intimacy among users [35,36], as well as how stories get promoted by Facebook’s algorithm [37]. Future public information campaigns on deemed consent for organ donation will need to better understand web-based public discourse and be better prepared to effectively disseminate accurate information while countering and correcting misinformation. Our study elucidated these precise issues for Facebook discussions as the new organ donation legislation and policy rolled out in Nova Scotia.

## Methods

### Overview

Our project examined web-based commentary around the deemed consent legislation changes produced on public (as opposed to private) Nova Scotia–based Facebook groups. To the best of our knowledge, no other study has examined a social media platform for the public’s web-based response to this new legislation. We chose to investigate Facebook as the platform has a significant social and demographically diverse influence [18,20,25,38], and intensive observation revealed Facebook to be the primary social media platform where most relevant discussions concerning Nova Scotia occurred. It is well known that Facebook groups represent a popular way for individuals to congregate, discuss, and share information [14,20]. A growing body of research shows that Facebook can be a source and propagator of misinformation [32], and several studies have demonstrated how web-based discourse, including Facebook comments, provides valuable insights into public perceptions and decision-influencing practices [13,14,39]. Therefore, we used comments and responses to posts in public Nova Scotia–based Facebook groups to analyze public perspectives on legislative change.

### Data Collection

We generated a sample of comments and replies for this study using the Facebook search function. We searched for posts in any public groups using the following inquires: ([“deemed consent” or “presumed consent” or “opt out” or “organ donation”] and “Nova Scotia,”) appearing between January 1, 2020, and May 1, 2021, extending from before the legislation came into force to the date the searches were performed.

All posts that appeared in public Nova Scotia–based Facebook groups were included in our data set. We did not include posts belonging to nationwide groups (such as the national Canadian Broadcasting Corporation) or groups affiliated with other provinces. As the *Results* section shows, our selected time frame encompassed the period of relevant public discussions, which occurred from late June to early July 2020 and again in mid-to-late January 2021, corresponding to when the legislative changes were implemented on January 18, 2021.

On May 25, 2021, we opened all the comments and replies on the respective Facebook post pages and took screenshots of all commentaries in discussions, saving this data in a Google Docs folder. All data were held in Google Docs folders accessible to all coders, and the analysis was conducted in shared Google Sheets.

The screenshots provided a fixed data set that could be subject to iterative analysis involving 3 coders over several months. Although usernames appeared in the screenshots, neither the usernames nor attributable accounts of individuals were included in the analysis to protect user privacy. For each post, we recorded the total counts of shares and the number of emoji reactions by type, which are *Like*, *Love*, *Care*, *Wow*, *Haha*, *Anger*, and *Sad*. Multimedia Appendix 1 provides visual images of these emojis.

### Coding and Analysis

We performed 2 analytic procedures on the data set to answer our two central research questions: (1) what perspectives did Facebook users express in comments about the new deemed consent organ donation legislation in Nova Scotia, and (2) how did Facebook users respond to the commentary of others? First, we used thematic analysis [40-42] as a means of capturing the wide range of public perceptions evident in the discussions. Second, we performed content analysis [43] on the 3 comments that garnered the most reactions in each discussion to provide insights into the kinds of comments resonating most strongly among the users.

Thematic analysis is a flexible qualitative approach that provides a highly detailed and complex summary of rhetorical data without sacrificing a plurality in meanings [40-42]. It has been used in other health contexts to analyze web-based commentary, including on Facebook [44,45]. This analytical method was well suited for the analysis of web-based comments derived from numerous socially diverse Facebook groups. This enabled us to obtain a detailed overview of the diverse themes, defined as “central organizing concepts” [41] as they appeared across disparate groups and at different periods.

Performing thematic analysis requires choosing between an “inductive approach,” where the data dictate the themes that emerge through analysis, and a “deductive approach,” where ideas, concepts, and themes are brought to the data before analysis [40]. We blended the 2 approaches as 2 coders knowledgeable on the topic brought some concepts, topics, and expectations to the study before engaging the data. However, the coders were not limited to these previously obtained perspectives as they anticipated, and were willing, to observe new rhetoric, topics, and language indicating emergent themes. The 3 coders followed the 6 phases of thematic analysis described in detail by Braun and Clarke [40] and examined the data for “trustworthiness,” as outlined by Nowell et al [42]. Careful attention was paid to constructing themes that were “specific enough to be discrete” but sufficiently broad to capture “ideas contained in numerous text segments” [42].

For the content analysis, we first determined the 3 comments that elicited the most emoji reactions in each discussion and then conducted the content analysis [43] on these comments. We applied the previously conducted thematic analysis categorizing each comment as pro (promoting or supporting the legislation or donation more broadly), critique (critiquing the legislation or donation more broadly), or neutral (reflections that neither clearly promote nor critique the legislation). We looked for any particular trends in the themes to provide greater insight into the comments that generated the most reactions from Facebook users. The content analysis was first performed by one coder, and a second coder checked all coding. There were only 3 disagreements between the coders, resulting in an intercoder reliability of Cohen κ=0.92, which demonstrates “almost perfect” levels of agreement [46]. The 3 discrepancies were resolved in a consensus session [47].

### Ethical Considerations

Ethics approval was not required for this research as the study involved analysis of publicly available data. The results do not contain any identifying information of commenters (eg, usernames), and the text examples have been paraphrased to further protect individuals’ privacy.

## Results

### Thematic Analysis

#### Overview

Our final data set included 26 posts with 2337 comments and replies from 12 different Facebook groups. Most comments appeared on Facebook groups belonging to either media companies or the Government of Nova Scotia. Some province-based community groups were also represented. The number of comments for each post ranged considerably (8-442; Table 1). The thematic analysis resulted in 4 principal themes and a total of 8 subthemes (Textbox 1). Textboxes 2-5 present each subtheme and illustrative excerpts from the Facebook comments.

**Table 1:** Complete data of Facebook groups and discussions^a^.

Facebook group name	Number of discussions	Number of comments	Date of discussions by post date
Nova Scotia Department of Health and Wellness	6	333	June 30, 2020; December 22, 2020; January 12, 15, 18, and 18, 2021
Nova Scotia Government	2	579	June 30, 2020; January 18, 2021
Nova Scotia Health	1	8	July 2, 2020
CBC Nova Scotia	3	462	July 1, 2021; January 18 and 28, 2021
Q97.7	1	104	January 19, 2021
Halifax Muslims	4	168	August 13, 2020; January 15, 15, and 17, 2021
Black NS News	1	13	December 18, 2020
Global Halifax	2	118	June 30, 2020; January 15, 2021
CTV Atlantic News	1	172	January 18, 2021
The Chronicle Herald	2	207	January 19 and 19, 2021
Halifax Today	2	152	July 15, 2020; January 19, 2021
Cape Breton Daily News	1	21	January 18, 2021

^a^Total: 26 discussions, 2337 comments; 2020—8 discussions; 2021—18 discussions.

**Principal themes and respective subthemes.**

**Supporting and promoting donation and transplantation and the new donation legislation (theme 1)**

- Caring about donation is caring about others
- The legislation isn’t a problem, and here’s why you naysayers are ignorant, stupid, selfish, and wrong

**Raising issues with donation and transplantation broadly and critiquing the new donation legislation (theme 2)**

- The legislation conflicts with my personal principles and world views
- They’re out to get us! They’re not to be trusted!
- Why fix what isn’t broken?! The changes aren’t needed or justified

**Discussing particulars and pointing out issues (theme 3)**

- Religious beliefs about donation and transplantation
- Is donation from gay men acceptable now?
- Family power is a benefit and a concern

**Metacommentary, softer reflections, jokes, and questions (theme 4)**

- Not applicable

**Paraphrased examples for theme 1 (supporting and promoting donation and transplantation and the new donation legislation).**

**Caring about donation is caring about others**

- Losing a child who was waiting for a transplant was horrible. Nova Scotia’s new initiative will be beneficial and “it’s about time” something like this was done.
- 100% support for the legislative change; many more organs will be available, and lives will be saved.
- A friend died in a tragic accident and their donated organs helped five different people. The donation was enormous gift, and the donation brought comfort to his grieving parents.
- Donation doesn’t just improve the quality of life for recipients but offers a means for grieving families to find comfort; donation provides hope to all.
- Waiting for organs is a serious struggle, and the new legislation is a splendid idea.

**The legislation isn’t a problem, and here’s why you naysayers are ignorant, stupid, selfish, and wrong**

- Those opting out should be ineligible to receive. “Selfish” people who don’t want to help shouldn’t get the chance to be helped.
- Having to check a simple box to opt out is not something to be upset about. It’s ridiculous and silly to think your rights are being “taken away.” Shut your whiny mouth
- Italy has done this for a long time, and many other jurisdictions should be like them.
- Those wanting to opt out are being “selfish Neanderthals”.
- Giving consent to donate is ok but having to consent to not donate is a “big deal”? It makes no sense to not help a dying child and just have the organs “rot instead.”
- It’s inexplicable why people are upset about this. Your organs are going “to rot in a hole” and there’s nothing science can do about it, so you may as well save someone else’s life.
- It’s a selfish position to not want to help save someone’s life, and it’s nonsensical why some see the new legislation as an issue.

**Paraphrased examples for theme 2 (raising issues with donation and transplantation broadly and critiquing the new donation legislation).**

**The legislation conflicts with my personal principles and world views**

- I agree with donation, and I am a donor, but I believe it is a decision for each person to make. It’s not right for others to “take” an organ unless you say no, and the new law acts as a “dangerous slippery slope.”
- The legislation is wrong, and the Nova Scotia government is not the owners of others’ bodies.
- Many other cultures, religions, and minorities care about how donation is done, and there is no clarity around how these processes will take place, especially as time is very sensitive in these contexts.
- It’s a serious cause of frustration as I support donation but disagree with the government taking ownership of a body after, say, a brain injury. The issue is that opting out is the only way to protect a right to choose.
- The government is treating us like “fucking lab rats,” robbing our graves, and assuming ownership over dead bodies!
- The new legislation is 100% WRONG! It’s a dangerous situation to have a law that “removes sovereignty” and seizing the ownership rights of a body that has not yet died.

**They’re out to get us! They’re not to be trusted!**

- People need to wake up and realize it’s about harvesting organs and selling them.
- They are trying to “trick people,” hoping people won’t know what’s happening.
- Come on over, Russia, take our body parts after the government takes away our firearms.
- It was “not cool” how MacNeil put this through secretly.
- The doctors will determine who is worthy to live and will let some die to save others’ lives. Say bye-bye to sick old people.
- It’s big business to sell body parts but now they know where you live and don’t even wait for you to die.
- Doctors don’t know about all rare diseases, and for some people they can prevent more problems by choosing to opt out.
- A nurse advised me once to not sign a donor card, and I think it’s because she saw a case of organs being taken before it was time for them to die. This legislation only works if people are lazy.
- This is “all about money.” Doctors are crooked and harvesting is a way to make money.

**Why fix what isn’t broken?! The changes aren’t needed or justified**

- The option already in place was “just a check mark,” and so if everyone agreed with being a donor why was the new law necessary? Why take away others’ rights?
- There was no reason to change the old way. Assuming is not right. Some, like myself, will be very confused by the opt out system.
- There is nothing about consulting the family, just that not opting out means the organs get taken. This new change will be expensive for taxpayers, and there is nothing wrong that needs fixing.

**Paraphrased examples for theme 3 (discussing particulars and pointing out issues).**

**Religious beliefs about donation and transplantation**

- Support for organ donation can be found in the majority of Muslim scholars.
- Loving God, who loves all children, means loving others and becoming organ donors.
- In my interpretation, if one’s body is not for one to decide whether it lives or dies, why should a person decide they can give it to others? On this basis, organ donation is not permitted, and Muslim organs donated to non-Muslim bodies will no longer be “cleansed.”
- Judaism frowns upon donation after death but not in live liver or kidney donations, but I understand that others choose to donate. For some cultures, giving away organs is like giving away part of the soul.
- Respect should be given for those who decided not to donate organs, whether that be for religious beliefs or other reasons. These people should not be attacked or called “monsters”; it’s too much.
- There is no reason to opt out except for being selfish and having “faith.”
- Some people can’t donate because of their religious beliefs.

**Is donation from gay men acceptable now?**

- I guess that now they will start accepting blood donation from me as a gay man?
- Prohibited from donating blood as a gay person in Nova Scotia, I guess that since a gay person’s blood isn’t acceptable, neither are the organs.
- Being gay means my organs can’t be donated.

**Family power is a benefit and a concern**

- The family veto issue is “pesky” as your next of kin’s wishes should be seen as YOUR wishes!
- The new legislation means that a family will be consulted by a nurse about donation if a person hasn’t registered a decision, and the family has the power to say no.
- I’m glad that this will be the default, but a family overriding the desires of a person is something they shouldn’t be able to do.
- I had the most terrible experience with doctors pressuring us to “carve up” my brother, and for that reason I hope families can play a role in the donation decision.

**Paraphrased examples for theme 4.**

**Principal theme 4: metacommentary, softer reflections, jokes, and questions**

- The number of donation arguments and opinions on the Internet is exhausting.
- It’s not right to pass judgement on others, given how personal and emotional the donation decision is.
- People are incapable of having “calm” discussions now, and I’ve been watching this get worse over the years.
- I am still unsure about my decision on this topic.
- My abused liver offers nothing to nobody!
- Is any person too old for donation?
- While I might sign up to be a donor, what happens in the case of my children? Can I overrule the choice I might have made for them?

#### Principal Theme 1: Supporting and Promoting Donation and Transplantation and the New Donation Legislation

##### Caring About Donation Is Caring About Others

These comments demonstrated compassion and portrayed donation and transplantation as practices to be respected, promoted, and encouraged. Several personal and emotional anecdotes regarding donation and transplantation success were included. Users often implicitly and explicitly expressed altruistic sentiments, stating that organs from deceased individuals should be shared with others, which the new legislation would facilitate, thereby saving more lives. These comments showed a desire to help people and encouragement for others, including other provinces, to adopt a similar approach. Some comments expressed feelings of pride in Nova Scotia’s initiative. This subtheme included reactions to critiques of donation and transplantation and typically expressed surprise, dismay, and disappointment at others’ lack of altruism. Permeating this commentary was an implied trust in health care systems and workers, including physicians (examples in Textbox 2).

##### The Legislation Isn’t a Problem, and Here’s Why You Naysayers Are Ignorant, Idiotic, Selfish, and Wrong

Commentary in this subtheme was distinctly more aggressive and argumentative than in the first subtheme. These comments were often replies to other users expressing concerns or issues with the legislation. Users voiced arguments, frequently with frustrated and angry tones, about why the legislation, and, broadly, donation, should be supported. It was commonly argued that the new legislation maintained choice and autonomy, that opting out would be easy, and that bodies with the potential to save lives could now be more readily used. For example, common references to dead bodies and organs “rotting in the ground” highlighted the perceived waste of a valuable resource in the absence of donation. A very common argument was that people who opt out should not be eligible to receive transplants.

Typical features in this subtheme included name-calling, labeling people who opt out as “selfish,” and suggesting detractors are unintelligent or mentally ill. Some users emphasized that deemed consent is relatively common for other legal procedures, such as with wills and estates, and is in place for donation in other countries. In a few cases, comments included statistics to support arguments (eg, the fact that an individual is much more likely to need a transplanted organ than to be an organ donor). Many comments attempted to contest and correct misinformation presented by others (examples in Textbox 2).

#### Principal Theme 2: Raising Issues With Donation and Transplantation Broadly and Critiquing the New Donation Legislation

##### The Legislation Conflicts With My Personal Principles and World Views

Central to this subtheme was the idea that powerful entities (namely, governments) were usurping individuals’ agency; acting against personal rights, autonomy, and freedom; overriding religious and spiritual beliefs and convictions; and diminishing people’s ability to consent. In numerous instances, users presented the concept of consent in absolute terms, suggesting that consent can and should not be presumed or negotiated (“my body, my choice”). Common arguments included the idea of the government assuming “ownership” of individuals’ bodies and the notion that this legislation was another example of the government increasingly encroaching on individual autonomy (eg, “slippery slope” and “what comes next?”). Tied into these sentiments was the idea that powerful entities would callously exploit bodies in undignified ways (“chopping up”), violating the perceived sanctity of the body and personal wishes upon death. Some users explicitly stated their desire to support organ donation while disapproving the new legislation. In a few cases, comments exhibited antialtruistic sentiments, such as explicitly stating their preference to not help others or to only help their family members (examples in Textbox 3).

##### They’re Out to Get Us! They’re Not to Be Trusted!

This subtheme centered on user comments, demonstrating a profound mistrust of the government and health care systems. These comments raised issues regarding the lack of transparency and consultation efforts of the government and health institutions. Common rhetoric included terms such as “tricky” and “secret” and phrases such as “hidden in legislation.” These users often argued that the government was intentionally (and maliciously) trying to dupe the public. Some comments directly targeted Nova Scotia’s then-governing Liberal Party and then-Premier Stephen McNeil (eg, labeling him a “dictator”). Some comments also disparaged the new legislation by comparing the changes with actions by foreign totalitarian governments.

As in the first subtheme, some users expressed concerns about the undesired exploitation and manipulation of bodies. However, such comments in the second subtheme underscored the nefarious objectives of public officials, including profit motives and sacrificing lives to save others (“harvesting”). Some comments suggested that increased organ procurement would cater to the needs of the rich (the poor would get worse service), take advantage of vulnerable populations (the homeless, youth, and those with mental health issues), involve transplanting infected and damaged organs unknowingly (eg, Lyme disease and HIV), result in fewer efforts to save lives to supply more donor organs, and cause data errors with serious consequences (eg, mishandling of individual health records). Many of these comments touched on conspiratorial ideas (examples in Textbox 3).

##### Why Fix What Isn’t Broken?! The Changes Aren’t Needed or Justified

This subtheme was characterized by an argumentative commentary about the need for a new model for donor consent. Users argued that if people wanted to donate, nothing in the old donation model would prevent them from doing so. Users also raised the parallel argument that the shortfall in donations was because people did not want to donate rather than merely forgetting or neglecting. In addition to questioning the legality of the new legislation (eg, “this won’t hold up in the courts”), users criticized the new model’s costliness. Comments typically argued that the old opt-in model was better—as it maintained personal choice and autonomy—and that the old model should instead be improved by, for instance, requiring the public to declare a donation preference when renewing a health card (examples in Textbox 3).

#### Principal Theme 3: Discussing Particulars and Pointing Out Issues

##### Religious Beliefs About Donation and Transplantation

Many of the discussions touched on religion; however, comments tended not to be specific to the new legislation. Users offered questions and observations about whether donation and transplantation align with the tenets of various religions, including concerns about donation conflicting with religious beliefs and the need to opt out for religious and spiritual reasons. It was uncommon for users to state their own religious convictions about donation. Rather, those commenting about religion typically generalized and assumed what others believed and felt. Such generalizations were often accompanied by the opinion that opting out for religious or spiritual reasons was an acceptable choice. Several users argued that specific religions, notably Islam and Christianity, allowed donation and transplantation and that refusing to donate might be contrary to principles of charity (examples in Textbox 4).

##### Is Donation From Gay Men Acceptable Now?

A few discussions raised the issue of whether donated organs from “gay” men would have specific restrictions, as with blood donation. Users raising this concern expressed offense at such discriminatory policies, although these critiques were not specific to the new legislation (examples in Textbox 4).

##### Family Power Is a Benefit and a Concern

Comments in this subtheme related to the power granted to family members to make donation choices on behalf of an incapacitated person. Some users expressed comfort in such a safeguard, whereas others expressed concern about family members overriding an individual’s decision (family veto). The importance of discussing donation decisions with one’s family was raised often (examples in Textbox 4).

#### Principal Theme 4: Metacommentary, Softer Reflection, Jokes, and Questions

The core characteristic of this theme was a neutral stance on the new legislation, which included reflections on the discussions, requests for information, and attempts at humor. These reflections discussed donation and transplantation, as well as thoughts on Nova Scotia and the nature of the modern media. Comments about donation and transplantation, and specifically the new legislation, included requests for clarification on facts and common practices (eg, eligibility to donate) and requests for the opt-out link. Some users questioned their eligibility to donate, in some cases making self-deprecating remarks about personal health and the unsuitability of their organs for donation (examples in Textbox 5).

### Content Analysis

Analysis of the top 3 comments with the most emoji reactions in each discussion (80/2337, 3.42%) demonstrated that positive emojis (*Like*, *Love*, or *Care*) were the most common, accounting for 95.45% (1112/1165) of all emoji reactions. Indeed, in the total sum of reactions (n=1165), negative reactions (*Anger* and *Sad*) only accounted for a small number (n=4, 0.34%). Multimedia Appendix 2 provides complete numbers. However, the types of comments that generated the nearly universal positive emoji reactions were a mix of responses to the new legislation or donation and transplantation broadly: positive (57/80, 71%), negative (13/80, 16%), and neutral (10/80, 13%; Multimedia Appendix 3). Thus, comments that were supportive, neutral, and critical toward the new legislation received positive emoji reactions from others.

The commentary that evoked the most positive reactions typically included both subthemes from theme 1, including one observable trend: 12% (7/57) offered a personal anecdote of donation and transplantation benefit, and 12% (7/57) exhibited an effort to correct misinformation. The oppositional commentary that provoked the most reactions was related to all 3 subthemes, including 2 with antialtruistic comments (not wanting to help others). The neutral commentary that garnered the most reactions was related to themes 3 and 4, including some discussions on religions and attempts at humor.

## Discussion

### Principal Findings

Nova Scotia is the first jurisdiction to pass deemed consent for organ donation in North America, and this study is one of the first studies to analyze web-based public discussions on the topic. The results of our analysis show that the new legislation generated controversy, with commentary displaying mixed reactions to the new legislation specifically and donation and transplantation broadly. A range of perspectives was expressed and fervently argued among Facebook group users. The principal themes that emerged from the analysis comprised being in favor and supportive, being opposed and critical, not being openly opposed or in favor but raising particular issues, and general commentary from a neutral perspective; some of these themes have been noted in the literature regarding deceased donation in general [48]. The subthemes constituting these principal themes, which touched on the topics of power, autonomy, government authority, religion and altruism, policy options, and argumentative strategy, provided key insights into how these diverse perspectives were supported and propagated, which is valuable for informing public outreach initiatives. These findings also demonstrate some key dynamics of user engagement with health policy news on social media.

### Findings of Public Perception in Other Contexts

Our research findings need to be contextualized through comparisons with legislated changes to organ donation consent in other jurisdictions. Nova Scotia joins England, Scotland, Wales, and Northern Ireland, which also recently moved to deemed consent models. National jurisdictions that have implemented deemed consent legislation variously observed increases and decreases in donation rates. Notably, Brazil saw a sharp decrease, as did Chile and Singapore [10]. Conversely, the Netherlands and Hong Kong both experienced increases in donation rates [10]. However, the general consensus is that modifying the consent model is not the key action generating an increase in donation rates [6,10,49]. Nevertheless, changing consent models can affect cultural norms and social consciousness, shifting the default position toward universal donation. Importantly, trust in the health care system and regional government is crucial to the adoption or rejection of donation policies, which includes how changes are communicated and how data are managed, especially as different contexts show that there are diverse public perceptions around implementing deemed or presumed consent models [10,48,50,51].

Similar research on public perceptions of deemed consent was recently conducted in Scotland, Northern Ireland, and England [52]. These researchers performed thematic analyses on free-text responses by individuals stating whether they would opt in or out or they remained unsure of the newly legislated donation scheme. The themes observed in that study corroborate our findings. Users who supported the switch to deemed consent also stressed how the new legislation promoted altruism and gave arguments about eligible body parts saving lives rather than being “wasted.” Our findings similarly revealed that these proponents included personal and emotional anecdotes about transplantation. Narrative messaging can have a powerful impact on how others perceive a range of issues [53,54]. As such, the sharing of positive personal anecdotes about donation and transplantation in public web-based spaces could be a valuable strategy for motivating others to consider donation [55]. Indeed, although not quantifiable, we observed that personal narratives shifted the tone of the discussions. In addition, our content analysis showed that some of the most liked comments were positive personal stories of donation and transplantation.

Further corroborating our findings, the UK study [52] showed that supporters of deemed consent stressed the idea of “reciprocity,” (ie, those willing to receive should be willing to donate) and commonly labeled people wanting to opt out as “selfish.” We speculated that users voicing such opinions—that individuals opting out should not be eligible to receive transplants—might have been motivated by reading others’ negative comments. We also considered name-calling and the labeling of detractors as “selfish” to be reactionary responses. However, contrary to our speculation, the UK study [51], as well as survey research in the United Kingdom [56], demonstrated that such sentiments constitute a core principle of equity for proponents of the policy. Therefore, any prospective change to opt-out consent models should acknowledge the potential for social tension to arise in the public discourse. Our study shows the tension between those who desire total public participation in donations and those who have reasons to opt out. The complexity of public perceptions and approaches has been previously reported in the literature [57]. Tension typically surfaces only with opt-out systems as opt-in models do not require people to actively or openly state their (perceived) opposition to donation. Rather, opt-in registries often do not require people to declare their donation preference, which seems to dilute this polemic.

The power of family members to veto an organ donor’s wishes or to grant final authority for donation appeared as an important subtheme in both our analysis and in the UK research [52,56]. Respondents who wished to explicitly state their opt-in position within the deemed consent model often believed that such a declaration would aid family decisions about donation and protect their personal choice from family interference. Our findings showed that commenters were both relieved and alarmed by the rights afforded to family members as ultimate decision-makers.

Policies addressing the involvement of family members are important for organ donation consent models. Trust in the health care system and in the organ donation process is paramount to any consent model being effective in increasing overall donation rates [10,48,49]. Prohibiting all forms of decision-making by family members would likely be perceived as inflexible and autocratic. Having frontline health care workers enforce a donor’s wishes against a family’s contestation is highly impractical and ethically problematic. Speaking to this issue in the Canadian context, an expert study asked whether it is “unrealistic to assume the next-of kin refusal rate would decrease under opt-out legislation” [49]. What factors and circumstances would foster a culture where family interference with donations would decrease? Careful monitoring and evaluation of the deemed consent program implementation in Nova Scotia will help answer these questions.

The salient themes we observed among users expressing concerns and grievances about deemed consent are also corroborated by the findings of the thematic analysis conducted in the United Kingdom [52]. Opponents in Nova Scotia emphasized a mistrust of the health care system and criticized the government for infringing on individual freedom and autonomy. Indeed, mistrust of the health care system is known to be a significant barrier to organ donation [10,49], especially given that opt-out policies can be perceived as deceitful, manipulative, and restrictive [58]. In both the UK study [52] and our study, opposition to deemed consent included personal beliefs about government power, philosophical views about consent, and practical concerns about organ donation procedures. For example, both studies found that opponents expressed worries about the unequal provision of health care services, the contested “ownership” of bodies or body parts, and the perceived uncertainty around declaring brain death for donors. However, despite many shared themes, only our study found critiques of the government for profiteering from excised organs. In addition, ideas around health care incompetence (eg, mistakenly transplanting diseased organs) were also seemingly unique to the Nova Scotian context.

### Acts of, for, and Against the Body

A pervasive feature of the Facebook discussions we analyzed was diverse and sometimes contrary perspectives associated with how the body is manipulated during organ donation and transplantation. The UK study [52] also observed similar issues. Both studies found that people addressed the body as something to be “used” or “recycled”—a valuable resource not to be “wasted.” In our research, the phrase “rot in the ground” was commonly used to both support the commenter’s prodonation position and criticize others for opting out (“wasting”). Interestingly, some of the most visceral, harsh, and argumentative language in our sample invoked the “rot” rhetoric. Some telling examples included comments about selfish people’s “useful organs” rotting and getting “eaten by bugs” and organs rotting away in “holes” instead of saving lives. This rhetoric is open to several interpretations—as an argumentative tactic, commentary on differing perspectives of the afterlife, a means of virtue signaling, or even a shocking reminder of the inevitability of death. Although this kind of rhetoric is forceful and abrupt, it is unlikely to constructively change the discussion or the perspectives of opponents of the organ donation legislation. If anything, it serves to exacerbate tensions.

Our interpretation of criticisms of organ procurement that referred to the body differed slightly from the UK team’s analysis [52]. For instance, we understood the “chopping up” rhetoric to suggest the undesirable and callous handling of bodies. The verb “chop” emphasizes the violent physicality of abuse occurring in organ removal and recovery. We also interpreted the often-repeated “harvesting” phrases to exemplify the impersonal corporeality described with terms such as “biopower,” which refers to the state exploiting bodies for governmental objectives [59]. Certainly, the concept of “biopower” could be applied to many of the objections raised by those opposing the legislation on the grounds of ownership, ethical consent, and state abuse among others. Similar to our study, the UK study [52] identified the subtheme of a “violation to bodily integrity,” which included participants who stressed a desire to have their bodies remain intact during and after death.

Unsurprisingly, much of the rhetoric about the body’s sanctity intertwined with religious topics. We observed discussions on how donation and transplantation aligned or conflicted with various theologies, including those grounded in Islam, Judaism, Protestantism, and Catholicism. Facebook users offered religious doctrines and observances as arguments both for and against organ donation. For example, users discussing Islamic beliefs debated the conflicting priorities of maintaining the “sanctity” of the intact body and offering organs to others as a charitable duty [60,61]. Some argued that donation is “frowned upon” in Judaism, whereas others stated that “loving God’s children” is well served through organ donation.

Research shows that religious beliefs can affect people’s perspectives on organ donation [62]; however, caution should be exercised when generalizing the influence of religion, especially with regard to opposing donation [61,63]. Indeed, many commenters in the Facebook groups assumed that some people would opt out because of religious beliefs. Similarly, survey research in the United Kingdom found that most believed organ donation to conflict with religious beliefs [56]. These perceptions about religious opposition to donation are in contrast to the fact that no major world religion has a total prohibition on organ donation; rather, organ donation is often connected to concepts of altruism and the ability to save lives [61]. The Facebook discussions we analyzed typically seemed respectful and supportive of people choosing to opt out because of religious beliefs, although it remains unknown how widespread the opt-out position is among religious communities. Effective public outreach should certainly account for the role that religion plays in promoting opinions about donation by engaging communities respectfully and proactively while fostering transparency in health care systems [61].

### Social Media and Misinformation

Analyzing web-based discourse provides insights into how these perspectives are expressed and propagated. Analyzing reactions via reaction tools (eg, *Likes*, *Loves*, and *Wows*) and reply chain discussions helps us understand how news posts and comments are received, debated, and refuted. In other words, how information is taken up, altered, and disseminated through these web-based spaces. Our research has some important media-related findings that are relevant to deemed consent donation laws and broadly to social media.

Most user comments appeared on Nova Scotia government Facebook groups, including the Nova Scotia Department of Health and Wellness (333/2337, 14.25% of all comments) and Nova Scotia Government (579/2337, 24.78% of all comments). These numbers demonstrate the value of government institutions using social media for public outreach, although other discussions might occur on private Facebook groups. There are benefits and challenges that arise with government entities hosting discussions. Hosting enables moderators from the organizations to analyze commentary; facilitate access and analysis from others; moderate discussions (deleting comments or blocking users if necessary); and respond to questions or comments, especially regarding misinformation.

In our study, moderators responded to questions about the donation and transplantation process broadly (eg, age limits for donation and transplant procedures), as well as specific aspects of the new legislation (eg, opt-out process and opt-out choices). Moderators, as well as other commenters, shared text and links to accurate websites, including the official government pages. Such information sharing is helpful to disseminate policy facts, for example, that people could opt out of donating some specific organs. Although not quantifiable in our research, we observed that moderator participation typically had a positive impact on discussions, especially when correcting misinformation or providing clarity around policies. However, as documented in other research, moderating poses numerous challenges, including how and when to engage commenters and on what grounds comments should be blocked or removed [64]. Ongoing research is to determine effective strategies that health practitioners and institutes can use in different web-based contexts [65].

The spread of misinformation through social media has been studied in a wide range of contexts. Information scholars have distinguished between misinformation and disinformation, where disinformation refers to an intent to spread inaccuracies and make facts appear ambiguous [66]. In this study, we observed numerous inaccurate comments. For example, some users suggested that the legislation was passed as a means of generating profits for the government and physicians. One such comment claimed that Canada is the leading exporter of heart valves and bone marrow, and the new legislation is focused on greed rather than helping fellow Canadians. Other comments stated that opting out was not possible after the legislation enactment date (January 18, 2021) and that the new legislation would not take into account the wishes of one’s family. Others stated that physicians would intentionally let people die to obtain organs for others. A significant number of these inaccurate comments were frequently repeated by the same few users. Although research projects have likened this activity of users repeating inaccuracies to disinformation [39,67], it is not possible to draw that conclusion in this context as the potential intent to deceive remains uncertain.

We observed numerous efforts to correct or debunk misinformation, especially in terms of the opt-out date and family involvement. Users variously countered misinformation with links to official government websites; personal expertise; and, when someone suggested that physicians will kill potential donors, by detailing the Hippocratic oath—known in Canada as the Code of Ethics and Professionalism [68]. Interestingly, we did not see much countering of the messages around profiteering from the selling of transplant organs. Ideally, there would have been some forthright messages clarifying these issues, especially from moderators or experts in the field.

When correcting misinformation, some scholars have raised concerns around the “back fire effect” [69], which argues that correcting misinformation leads to increased adherence to the misinformation. However, such an effect and concern do not appear substantiated in research [70]. There is certainly value in attempting to correct misinformation and promoting accuracy [71,72], especially in this specific context, for more uncontestable facts (eg, a deadline to opt out). Indeed, our content analysis on the most reacted-to comments showed that some of the most liked comments included debunking efforts. Users liking the debunking comments indicated that many participants valued their contributions to the discussions. However, what remains unclear is whether those comments had any influence on those who disagreed with the sentiments expressed. Although we did not perform an analysis on the different users participating in discussions, it would seem highly valuable for experts to weigh in on public discussions and provide clarity and accurate information whenever possible. Indeed, research specifically on Facebook has also shown how comments from experts receiving a relatively high number of likes are perceived as the most credible health messages [73].

Those few especially vocal commenters who spread inaccurate or conspiracy-tinged comments occasionally received backlash from other users in the group. In addition to correcting observed inaccuracies, some commenters also made concerted efforts to add accurate statistics to the discussions. For example, a commenter shared the statistic that a person is 6 times more likely to need an organ than to be an eligible donor. There were numerous instances where such a statistic might have usefully grounded abstract or polemic discussions. Indeed, new research has also argued that rather than countering misinformation, more effective public engagement would work toward improving the acceptance of reliable information [74]. This underscores the need for ongoing research to better understand how accuracy on the web can best be promoted, including a social media design that promotes critical reflection, and how public health agencies can productively engage the public on the web when dealing with polemic issues [66,72,75].

The Canadian Donation and Transplantation Research Program recently held a web-based workshop about public engagement on the web. Individuals from 3 health science organizations discussed their web-based public engagement strategies [76]. The workshop featured presentations from representatives of the United Kingdom’s National Health Services, Nova Scotia Health, and a new Canadian-based initiative—Science Up First—which aims to educate the public and debunk misinformation on a range of health and science topics. The presenters from the National Health Services and Nova Scotia Health spoke on the topic of deemed consent organ donation legislation, whereas the presenter from Science Up First spoke on their public engagement efforts during the COVID-19 pandemic. Key messages from all presenters highlighted the need for careful monitoring of web-based conversations and carefully planned strategies to tackle the presence of misinformation. Importantly, they noted that ongoing monitoring of conversations allowed moderators and communicators to track the kinds of problematic sentiments shared, as well as accounts that, in some cases, needed to be blocked or banned from further participation. In the context of deemed consent in Nova Scotia specifically, web-based moderators encouraged dialog among participants and only engaged to provide clarity and accuracy (often by providing links to government web pages) or to remove comments and, in a few selective cases, ban users.

Vital to these efforts was the creation of a detailed frequently asked questions document used to train moderators, equipping them with the tools to answer questions in a timely manner. A carefully constructed terms of use policy document, which participants might not read but which moderators can provide as evidence to an offending individual who had comments removed or who was banned from a platform for breaking its rules (eg, using abusive language and repeated offenses), is also essential. Importantly, moderators chose to provide accuracy and clarity when encountering misinformation—as opposed to deleting comments—to make people feel that their voices and concerns were being heard. Indeed, moderators and provincial health officials often positively interpreted web-based debates as public engagement on the topic and as engagement that ultimately reached bigger audiences and raised more awareness about the legislative change. The presenter from Nova Scotia Health also stressed the need for moderators to engage in a neutral, emotionless tone, which would help maintain the focus on relaying accurate information. All presenters noted that countering misinformation was likely less effective as a means of changing the perspective of those sharing inaccuracies but very valuable to help stop the viral spread and influence of misinformation on the wider audience. All organizations stressed the need to engage diverse communities and build wide networks that collaboratively work toward transferring accurate information and heightening science and policy literacy.

### Limitations

Our study analyzed comments in Nova Scotia Facebook groups during a relevant period using an approach consistent with research on similar objectives [52,77]. However, there are some limitations to consider when assessing this research. This project only analyzed comments in publicly accessible Facebook groups, and the demographics of those contributing comments are unknown. Therefore, the findings are not generalizable to the public. Analyzing user accounts can provide additional insights but raises ethical issues related to privacy and personal information. In addition to our analysis, conversations about this new legislation on private Facebook groups or even other social media sites might support or contradict our findings.

Although our analysis is particular to Nova Scotia, there are many similarities with research conducted in the United Kingdom [52]. As research on this topic continues to grow, we can better anticipate how the public in different jurisdictions might respond to similar legislation, thus enhancing the ability of policy makers and communication strategists to craft effective public outreach and engagement in policy and legislative reform.

### Conclusions

Facebook users in public groups expressed diverse and passionate perspectives about the new deemed consent organ donation legislation in Nova Scotia. These perspectives touched on the topics of health care systems, communities, government authority, religions, the body, death, and the afterlife. Critical perspectives need to be corroborated with other research on public perspectives and actual opt-out rates. Notably, since the implementation of presumed consent, Nova Scotia has experienced an increase in tissue donation and organ referrals while data show that only 5.7% of residents have opted out [78]. The degree to which the increases can be attributed to the organ donation legislative changes requires further and ongoing examination.

Even if concerns around deemed consent are held by a small minority, the issues should not be ignored. Trust is an integral component of health care systems and needs to be maintained and strengthened wherever possible. In the Canadian context, it is well known that mistrust circulates among racialized communities, notably indigenous communities, which have been subjected to colonization and systemic racism. Listening to the concerns of these voices and addressing concerns with actions can only help improve trust in the health care system. Some of these efforts include engaging individuals and communities in dialog offline and on the web. A proactive approach involves listening to issues, clarifying doubts where possible, providing transparency regarding policies, and correcting misinformation. Social media is a hotbed of misinformation; however, it also has the potential to effectively inform the public through creative and accurate messaging.

## Appendix

Multimedia Appendix 1 A total of 7 Facebook emoji reactions.

Multimedia Appendix 2 Complete number of top 3 comments in each discussion based on the number of emoji reactions.

Multimedia Appendix 3 Sentiment expressed in 3 most reacted-to comments (n=80) in each discussion (n=26).
