# Supplementary material for: Web-Based Perspectives of Deemed Consent Organ Donation Legislation in Nova Scotia: Thematic Analysis of Commentary in Facebook Groups
Source: JMIR Infodemiology. 2022 Sep 14;2(2):e38242. doi: 10.2196/38242 (PMC9987187; doi:10.2196/38242)
Supplement: Multimedia Appendix 2 [file infodemiology_v2i2e38242_app2.docx]

**Complete number of top 3 comments in each discussion based on the number of emoji reactions**

| Discussion | Total emoji reactions | Total positive | % | Total neutral | % | Total negativity | % |  | Pro comment | Neutral comment | Negative comment |
| --- | --- | --- | --- | --- | --- | --- | --- | --- | --- | --- | --- |
| 1 | 49 | 45 | 91.84% | 4 | 8.16% |  | 0.00% |  | 2 | 0 | 1 |
| 2 | 14 | 14 | 100.00% |  | 0.00% |  | 0.00% |  | 2 | 0 | 1 |
| 3 | 22 | 21 | 95.45% |  | 0.00% | 1 | 4.55% |  | 2 | 0 | 1 |
| 4 | 9 | 7 | 77.78% | 2 | 22.22% |  | 0.00% |  | 2 | 1 | 1 |
| 5 | 19 | 19 | 100.00% |  | 0.00% |  | 0.00% |  | 3 | 0 | 0 |
| 6 | 12 | 12 | 100.00% |  | 0.00% |  | 0.00% |  | 3 | 0 | 0 |
| 7 | 114 | 114 | 100.00% |  | 0.00% |  | 0.00% |  | 3 | 0 | 0 |
| 8 | 76 | 72 | 94.74% | 4 | 5.26% |  | 0.00% |  | 3 | 0 | 0 |
| 9 | 7 | 7 | 100.00% |  | 0.00% |  | 0.00% |  | 3 | 0 | 0 |
| 10 | 67 | 65 | 97.01% | 1 | 1.49% | 1 | 1.49% |  | 2 | 1 | 1 |
| 11 | 72 | 72 | 100.00% |  | 0.00% |  | 0.00% |  | 3 | 0 | 0 |
| 12 | 116 | 102 | 87.93% | 13 | 11.21% | 1 | 0.86% |  | 1 | 2 | 0 |
| 13 | 45 | 45 | 100.00% |  | 0.00% |  | 0.00% |  | 2 | 0 | 1 |
| 14 | 9 | 9 | 100.00% |  | 0.00% |  | 0.00% |  | 1 | 2 | 0 |
| 15 | 20 | 20 | 100.00% |  | 0.00% |  | 0.00% |  | 1 | 2 | 0 |
| 16 | 19 | 19 | 100.00% |  | 0.00% |  | 0.00% |  | 3 | 0 | 0 |
| 17 | 36 | 36 | 100.00% |  | 0.00% |  | 0.00% |  | 2 | 1 | 0 |
| 18 | 11 | 11 | 100.00% |  | 0.00% |  | 0.00% |  | 1 | 0 | 2 |
| 19 | 50 | 48 | 96.00% | 2 | 4.00% |  | 0.00% |  | 3 | 0 | 0 |
| 20 | 63 | 62 | 98.41% | 1 | 1.59% |  | 0.00% |  | 3 | 0 | 0 |
| 21 | 106 | 94 | 88.68% | 12 | 11.32% |  | 0.00% |  | 2 | 0 | 1 |
| 22 | 121 | 121 | 100.00% |  | 0.00% |  | 0.00% |  | 3 | 0 | 0 |
| 23 | 4 | 2 | 50.00% | 2 | 50.00% |  | 0.00% |  | 2 | 0 | 1 |
| 24 | 16 | 13 | 81.25% | 3 | 18.75% |  | 0.00% |  | 1 | 0 | 2 |
| 25 | 55 | 54 | 98.18% |  | 0.00% | 1 | 1.82% |  | 2 | 0 | 1 |
| 26 | 33 | 28 | 84.85% | 5 | 15.15% |  | 0.00% |  | 2 | 1 | 0 |
|  | 1165 | 1112 | 95.5% | 49 | 4.21% | 4 | 0.34% |  |  |  |  |
|  |  |  |  |  |  |  |  |  | 57 | 10 | 13 |
|  |  |  |  |  |  |  |  |  | 71.25% | 12.50% | 16.25% |
