# Supplementary figures and images for: Web-Based Perspectives of Deemed Consent Organ Donation Legislation in Nova Scotia: Thematic Analysis of Commentary in Facebook Groups
Source: JMIR Infodemiology. 2022 Sep 14;2(2):e38242. doi: 10.2196/38242 (PMC9987187; doi:10.2196/38242)

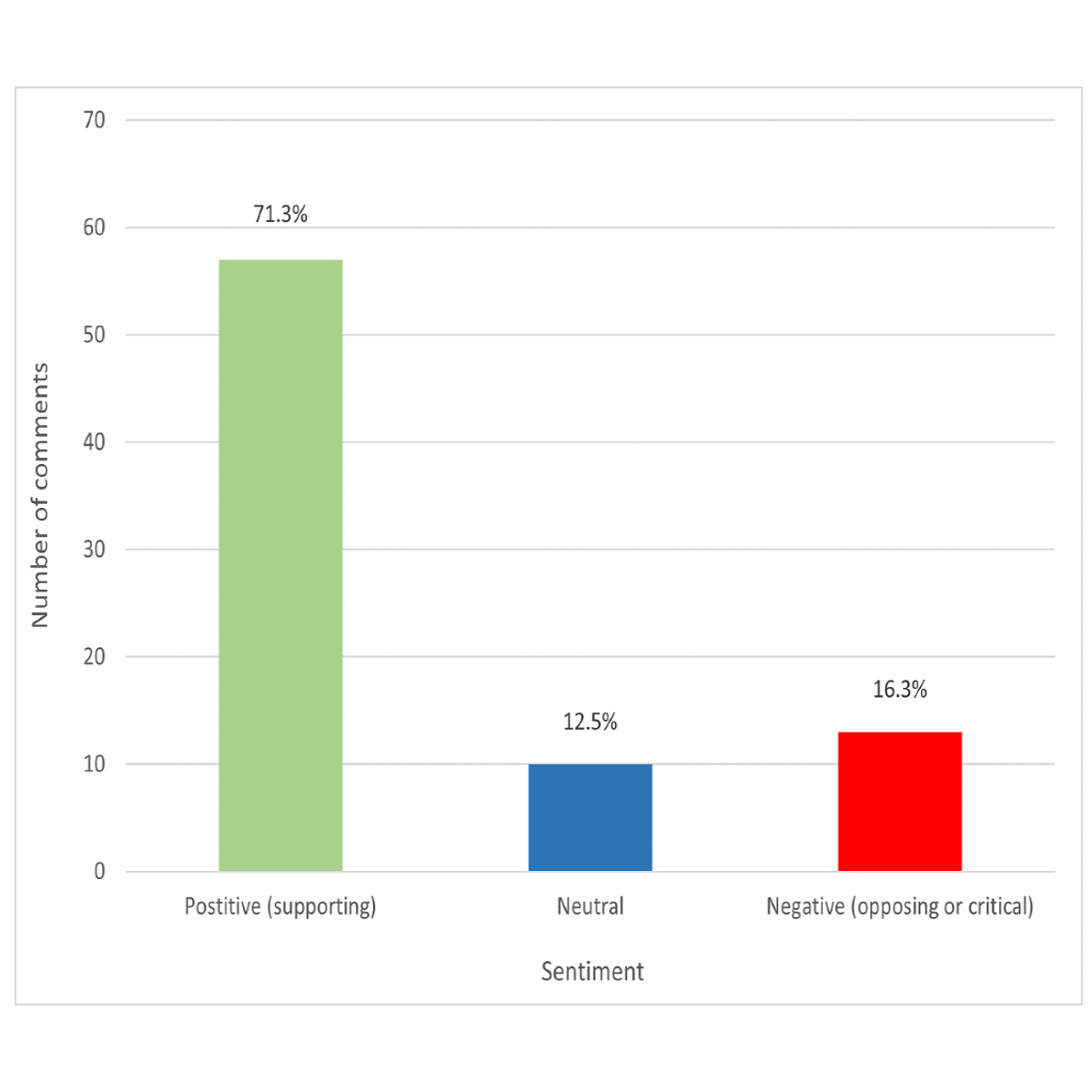

Supplement: Multimedia Appendix 3 [file infodemiology_v2i2e38242_app3.png]
